# Improving operating room safety

**DOI:** 10.1186/1754-9493-3-25

**Published:** 2009-11-20

**Authors:** Scott N Hurlbert, Jill Garrett

**Affiliations:** 1 Memorial Health System, 1400 E. Boulder St. Colorado Springs, CO 80909, USA

## Abstract

Despite the introduction of the Universal Protocol, patient safety in surgery remains a daily challenge in the operating room. This present study describes one community health system's efforts to improve operating room safety through human factors training and ultimately the development of a surgical checklist. Using a combination of formal training, local studies documenting operating room safety issues and peer to peer mentoring we were able to substantially change the culture of our operating room. Our efforts have prepared us for successfully implementing a standardized checklist to improve operating room safety throughout our entire system. Based on these findings we recommend a multimodal approach to improving operating room safety.

## Background

Memorial Health System is a community health care organization located in Colorado Springs, Colorado. It is comprised of 650 beds located across 2 campuses. There are 17 operating rooms located in the central campus and 8 operating rooms at the north campus. Approximately 18,000 surgeries are performed in the system in a single year.

## History

Our journey towards improved operating room safety started in June 2005. At that time we applied for and obtained a $50,000 grant sponsored by the Association of Perioperative Registered Nurses (AORN) with funding provided by Kimberly-Clark to introduce human factors training in the operating room. AORN was responsible for approving and administering the grant. Human factors training is based on the Crew Resource Management (CRM) programs championed by the airline industry. In the 1970s the airline industry was plagued by multiple high profile accidents that were a direct result of a toxic culture in the cockpit. Many of the same attitudes that were present during these dark days of the airline industry are currently present in the operating rooms of today. Before CRM the flight team was often afraid to challenge the captain even in the face of critical errors. Today in most operating rooms the staff also find it hard to question decisions made by the surgeon even though the decision may lead to patient harm. The working environment in both of these industries are characterized by significant on-time pressures, high workloads, dependence on properly working equipment, a rigid hierarchy, and a potential for catastrophic results if errors occur. Effective communication is critical for safety in both industries. The goal of these programs was to reduce the errors that occur from well-intentioned, highly skilled professionals working in a stressful environment.

## Methods

The money from the grant was used to subsidize human factors training for the OR staff and surgeons of our operating rooms. We used the company Safer Healthcare to provide the training. Throughout November and December of 2005 training sessions were held. Each of these training sessions lasted 4 hours. Physicians and operating room staff members were trained together to emphasize the team concept. The training was mandatory for the operating room staff, but voluntary for the surgeons. 200 perioperative staff and 60 physicians participated in the training. At the core of the human factors training was a preoperative briefing by the attending surgeon. This briefing is very similar to the checklists currently being proposed by the World Health Organization (WHO) [1]. The preoperative briefing sets expectations as to how the conduct of the case will proceed. It informs the operating room staff as to what equipment will be needed and if any difficulties are expected. More importantly the preoperative briefing also opens the lines of communication and helps to break down the hierarchy of the operating room. Under conditions of great stress it is easy to lose situational awareness and become focused on only one aspect of the case. Often there are other people in the room who recognize that an error is being made, but are too afraid to speak up. The preoperative briefing should encourage anyone in the room to speak up if an error is being made. A postoperative debriefing was also encouraged to help critique the conduct of the case. We measured two outcomes.

The first outcome we looked at was if the preoperative briefing resulted in any change in operating room culture. We used a survey from the Agency for Health Care Research and Quality (AHRQ) to measure the change. The other outcome was whether or not operating room efficiency and miscommunication events were improved with a preoperative briefing. Essentially we stationed an observer in an operating room throughout the day. They kept track of the number of times the circulating nurse had to leave the room to get equipment that had not been planned for. They also looked at miscommunication events and how that impacted on the conduct of the case. Specifically questions were asked how these events affected the dynamics of the team, whether the events adversely affected the conduct of the case, whether the events impacted what equipment was available, and whether or not the patient was adversely affected by the events. We compared surgeons who did briefings with surgeons who did not do briefings.

## Results

The initial human factors training was open to all of the surgeons who practiced at our hospital. The surgeons who participated in the initial training represented all of the major surgical fields. There was some concentration in general surgery, orthopedic surgery and vascular surgery just from the number of these physicians who practice these specialties at our hospital. Almost all of the surgeons were independent practitioners and not hospital employees. There were 60 surgeons who underwent the training.

At the beginning of the program there were 2 physicians who routinely did preoperative briefings. The human factors training resulted in another 20 physicians who routinely conducted the briefings. Most of the surgeons involved in the training saw the value in the briefing but didn't change their operative routine. The most common reason given by the surgeons as to why they didn't change was the perception that a briefing would slow down the progress of the case. Over the next 2 years the number of physicians remained relatively stable. The program remained completely voluntary on the part of the physicians. There were no other training classes provided. Instead we focused on peer to peer efforts to spread the message. The operating room staff was also encouraged to ask for briefings from the attending surgeons. Initially the major barrier to participation was that the physicians did not believe that doing a preoperative briefing would enhance their practice or patient care to any measurable amount. We also ran into some resistance from surgeons who felt that the whole human factors training was just another way for the hospital to try and control them.

**Figure 1:**
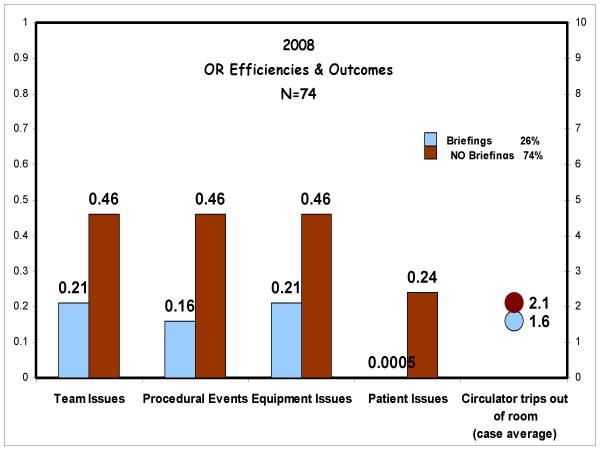
**Operating room efficiencies study**. These are the results of our local operating room efficiency study. The x axis is the issues studied. The y axis is the frequency of the issues expressed as a factor of 1. The light color represents the efficiency of the operating rooms that performed a preoperative briefing and the dark color represents the operating room that did not have a preoperative briefing.

By the beginning of 2008 it became evident that we needed to be more aggressive in our efforts to recruit doctors to do briefings. We invited Dr. Thoralf Sundt, a cardiothoracic surgeon at the Mayo Clinic to give a single presentation on how preoperative briefings have affected his practice. We also started a study in our operating room looking at miscommunication events and operating room efficiency. The results of this study are provided in figure 1. We found that there was a positive difference in the rooms that had a preoperative briefing. The briefings decreased the number of times the circulating nurse left the operating room. There was a rough correlation with the duration of the operation and the number of times the nurse had to leave the room. Patient issues were defined as any questions about the patient that should have been known ahead of time. For example if the patient was a diabetic did the anesthesiologist know this ahead of time. Team issues were defined as any miscommunication between the members of the operating room staff that resulted in a delay or adverse event. Equipment issues were defined as any time the appropriate instrument or device was not available at the time it was needed. A procedural event was any adverse event that affected the patient's care. This event did not necessarily need to be clinically significant. We did not specifically isolate how much time each issue cost the patient in terms of efficiency as this was extremely variable. Efficiency was measured indirectly as it was assumed that an operating room with fewer disruptions was more efficient. It is difficult to compare operating room times across different specialties and procedures. Since we had data on a local level we could show surgeons how a change in their behavior can positively affect the conduct of the operating room.

In February of 2008 a retired pediatric surgeon was hired to help coach surgeons on how to do a briefing. This surgeon would circulate among the rooms observing cases. She interacted with surgeons on a one-to-one basis providing guidance and advice on the best way to do a briefing. Her presence also was a reminder to do a briefing. She was present for approximately 3 months.

**Figure 2:**
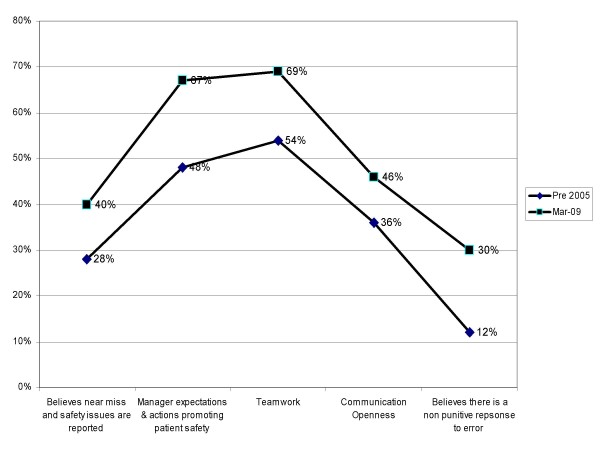
**Agency for Health Care Research and Quality Survey results for operating room safety before and after human factors training**. These are the Agency for Health Care Research and Quality Survey results of operating room culture before and after human factors training. The x axis are the specific questions asked. The y axis is the frequency of a positive answer expressed as a percentage. The dark line with the triangular data points represents the results of the survey done before human factors training. The dark line with the square data points represents our most recent results.

The combination of a single conference devoted to preoperative briefings, a local study demonstrating increased OR efficiency, and hiring a physician coach resulted in an increase in the number of surgeons doing briefings. Once we could show surgeons how care is improved with the briefings, it removed some of the skepticism over the process. By the end of 2008 48 surgeons were doing preoperative briefings. Throughout this time we were also conducting periodic cultural surveys. Over time the use of briefings has made our operating rooms a less hostile environment although we still have significant work to do. Figure 2 documents the improvement in operating room culture from before training and preoperative briefings to the present time. Specifically we found that as more and more surgeons did briefings the operating room staff felt that there was more teamwork and openness in communications than previous. We also found that the staff felt that there was less of a punitive reaction to errors. Overall the staff thought that the operating room was less hostile because of the briefings.

As of May 2009 54% of the over 6000 cases done at our system have had a preoperative briefing. It is important to note that a preoperative briefing is still entirely voluntary on the part of the physician.

At the beginning of 2009 the World Health Organization endorsed the implementation of a surgical checklist to help improve the safety of operating room.

The surgical checklist and the preoperative briefings are essentially striving for the same goal. We have used the surgical checklist as a way to further the use of the surgical briefing. Our hospital system is currently in the process of developing its own surgical checklist. We are incorporating many of the lessons that we learned from the preoperative briefing process into this new initiative.

## Discussion

Our journey to improving the safety of our operating rooms has been enlightening. The most important lesson that we learned is that initiatives for cultural change within the operating room have to be physician led. Without a core group of physician champions to lead the change the process becomes much more difficult. This is particularly true in a setting where a majority of the medical staff is independent. It was also important to avoid the perception that a preoperative briefing was something imposed by the hospital administration. In the current environment of increasing regulation physicians are becoming very sensitive to anything that is perceived as restraining their practice of medicine. In spite of having physician leadership we still were met with resistance to this change. We had about a year where not much progress was made. Peer to peer interaction was not enough.

Formal training to include all members of the operating room team was also essential. Doctors, nurses, and other operating room staff overall trained as a combined group to help foster a team approach. This also helped to break down some of the hierarchy present in the operating room. The nurses and other operating room staff became more comfortable in questioning the physician if they felt that something was going wrong. This was further strengthened in the operating rooms that had preoperative briefings. Because of funding limitations, only one formal training session occurred. More training may have increased participation.

Persistent and frequent reinforcement of the concepts that we learn with the human factors training was also important. Our number of briefings increased once we had a physician mentor in the operating room to help facilitate the briefing process. The physician mentor was a constant presence in the operating room to remind surgeons to do the briefing as well as to help the surgeon figure out the most efficient use of the briefing. This role should be held by a surgeon. Another type of physician would not be as effective as they do not have the same experience as a surgeon.

Besides formal training sessions, periodic guest speakers also keep the concepts fresh in everybody's mind and reinforce the importance of doing the briefings. These also allow physicians to see how outside facilities manage the briefings. We only had one outside speaker come and talk to us during the program. In the future having speakers present on a quarterly basis would be extremely helpful.

Finally having our own data to show physicians the actual benefits in safety and efficiency was crucial. These data were able to show the 'real world' affects of preoperative briefings. A common complaint that we hear from surgeons is that data obtained at other institutions are not valid for our own because of regional variation. Having a study done on the premises that shows a positive correlation with the preoperative briefing is very powerful in refuting this concern. We are continuing to monitor our operating room culture with the AHRQ survey. In the future we also hope to conduct another operating room efficiency study.

The program remained voluntary throughout its course. There are both strengths and weaknesses to this. The major downside to having a voluntary process is that cultural change is very slow. The fastest way to achieve 100% compliance is to mandate it throughout the entire operating room. This can engender a considerable amount of resentment from the medical staff. Passive and active resistance would be significant. Throughout our hospital system we're attempting a cultural change that involves more physician input into decision-making processes. We felt that imposing a set of guidelines on surgeons would actually hamper us from affecting any meaningful cultural change. The fact that the preoperative briefing is voluntary allows the surgeon to make the process their own. While overall acceptance is slower we believe that adherence to the principles behind the briefing will be more robust if every surgeon claims ownership.

We still have a considerable ways to go on our journey to improve operating room safety. The work we have done has laid a good foundation for developing a surgical checklist. We will use the lessons learned from this project to continue to grow our culture of safety. Fortunately others have also embarked on this journey and we can use their examples to help guide us [2-14].

## Conclusion

Operating room safety has a significant influence on patient care. We found that we needed multiple approaches to advance a culture of patient safety. Ultimately the best process occurs when physicians take ownership of the cultural change. We recommend that whatever approach systems take to implementing a culture of safety in the operating room that physicians are intimately involved in the process.

## Competing interests

The authors declare that they have no competing interests.

## Authors' contributions

SH was involved in study design and manuscript preparation. JG was involved in study design and data gathering. All authors read and approved the final manuscript.

## Authors' information

SH is a vascular surgeon. He was an early champion of the preoperative briefing and is currently Chief of Staff Elect at Memorial Health System.

JG is the perioperative care manager for Memorial Heath System. She wrote the grant and was responsible for its implementation.

## References

[B1] World Alliance for Patient Safety WHO guidelines for safe surgery 2009 Geneva: World Health Organization 23762968

[B2] Awad S Fagan S Bellows C Albo D Green-Rashad B De La Garza M Berger D Bridging the communication gap in the operating room with medical team training Am J Surg 2005 190 770 774 10.1016/j.amjsurg.2005.07.018 16226956

[B3] Haynes AB Weiser TG Berry WR Lipsitz SR Breizat AHS Dellinger EP Herbosa T Joseph S Kibatala PL Lapitan MCM Merry AF Moorthy K Reznick RK Taylor B Gawande AA A Surgical Safety Checklist to Reduce Morbidity and Mortality in a Global Population NEJM 2009 360 491 499 10.1056/NEJMsa0810119 19144931

[B4] Henrickson SE Wadhera RK ElBardissi AW Wiegmann DA Sundt TM Development and Pilot Evaluation of a Preoperative Briefing Protocol for Cardiovascular Surgery J Am Coll Surg 2009 208 1115 1123 10.1016/j.jamcollsurg.2009.01.037 19476900 PMC4282162

[B5] Kao LS Thomas EJ Navigating Towards Improved Surgical Safety Using Aviation-Based Strategies J Surg Res 2008 145 327 335 10.1016/j.jss.2007.02.020 17477934

[B6] Lingard L Espin S Rubin B Whyte S Colmenares M Baker GR Doran D Grober E Orser B Bohnen J Reznick R Getting teams to talk: development and pilot implementation of a checklist to promote interprofessional communication in the OR Qual Saf Health Care 2005 14 340 346 10.1136/qshc.2004.012377 16195567 PMC1744073

[B7] Lingard L Espin S Whyte S Regehr G Baker GR Reznick R Bohnen J Orser B Doran D Grober E Communication failures in the operating room: an observational classification of recurrent types and effects Qual Saf Health Care 2004 13 330 334 10.1136/qshc.2003.008425 15465935 PMC1743897

[B8] Lingard L Regehr G Orser B Reznick R Baker GR Doran D Espin S Bohnen J Whyte S Evaluation of a preoperative checklist and team briefing among surgeons, nurses, and anesthesiologists to reduce failures in communication Arch Surg 2008 143 12 18 10.1001/archsurg.2007.21 18209148

[B9] Makary MA Holzmueller CG Thompson D Rowen L Heitmiller ES Maley WR Black JH Stegner K Freischlag JA Ulatowski JA Pronovost PJ Operating room briefings: working on the same page Jt Comm J Qual Patient Saf 2006 32 351 355 16776390 10.1016/s1553-7250(06)32045-4

[B10] Makary MA Mukherjee A Sexton JB Syin D Goodrich E Hartmann E Rowen L Behrens DC Marohn M Pronovost PJ Operating room briefings and wrong-site surgery J Am Coll Surg 2007 204 236 243 10.1016/j.jamcollsurg.2006.10.018 17254927

[B11] Makary MA Sexton JB Freischlag JA Holzmueller CG Millman EA Rowen L Pronovost PJ Operating room teamwork among physicians and nurses: teamwork in the eye of the beholder J Am Coll Surg 2006 202 746 752 10.1016/j.jamcollsurg.2006.01.017 16648014

[B12] Sexton JB Makary MA Tersigni AR Pryor D Hendrich A Thomas EJ Holzmueller CG Knight AP Wu Y Pronovost PJ Teamwork in the operating room: frontline perspectives among hospitals and operating room personnel Anesthesiology 2006 105 877 884 10.1097/00000542-200611000-00006 17065879

[B13] Soar J Peyton J Leonard M Pullyblank AM Surgical safety checklists BMJ 2009 338 b220 b220 10.1136/bmj.b220 19158173

[B14] Undre S Healey A Darzi A Vincent C Observational assessment of surgical teamwork: a feasibility study World J Surg 2006 30 1774 1783 10.1007/s00268-005-0488-9 16983480

